# X Chromosome Sites Autonomously Recruit the Dosage Compensation Complex in *Drosophila* Males

**DOI:** 10.1371/journal.pbio.0020341

**Published:** 2004-10-05

**Authors:** Delphine Fagegaltier, Bruce S Baker

**Affiliations:** **1**Department of Biological Sciences, Stanford UniversityStanford, CaliforniaUnited States of America

## Abstract

It has been proposed that dosage compensation in *Drosophila* males occurs by binding of two core proteins, MSL-1 and MSL-2, to a set of 35–40 X chromosome “entry sites” that serve to nucleate mature complexes, termed compensasomes, which then spread to neighboring sequences to double expression of most X-linked genes. Here we show that any piece of the X chromosome with which compensasomes are associated in wild-type displays a normal pattern of compensasome binding when inserted into an autosome, independently of the presence of an entry site. Furthermore, in chromosomal rearrangements in which a piece of X chromosome is inserted into an autosome, or a piece of autosome is translocated to the X chromosome, we do not observe spreading of compensasomes to regions of autosomes that have been juxtaposed to X chromosomal material. Taken together these results suggest that spreading is not involved in dosage compensation and that nothing distinguishes an entry site from the other X chromosome sites occupied by compensasomes beyond their relative affinities for compensasomes. We propose a new model in which the distribution of compensasomes along the X chromosome is achieved according to the hierarchical affinities of individual binding sites.

## Abstract 2

Evidence is presented that the existing model for dosage compensation in *Drosophila* is incorrect, and a new model is proposed

## Introduction

Most X chromosomal genes are essential or relevant to both sexes. To cope with the difference in the number of copies of these genes in females (XX) and males (XY), organisms have evolved a variety of mechanisms, collectively termed dosage compensation, to equalize the levels of X-linked gene products in the two sexes. In *Drosophila* males the expression of most of the genes on the single X chromosome is doubled. At least six protein-coding genes, collectively referred to as *male specific lethal*s (*msl*s), are required for dosage compensation (Baker et al. 1994; Marin et al. 2000; Meller 2000): *msl-1, msl-2,* and *msl-3,* whose functions remain unknown; *maleless (mle),* encoding an RNA helicase; *males absent on the first (mof),* encoding a histone acetyltransferase; and *jil-1,* encoding a histone kinase. The products of these genes, together with noncoding RNAs encoded by the *RNA on the X* genes*(roX1* and *roX2)* (Amrein and Axel 1997; Meller et al. 1997; Franke and Baker 1999), are all reproducibly associated with hundreds of locations along the length of the polytenized salivary gland X chromosome in males. MOF has been shown both in vivo and in vitro to acetylate H4Lys16, a specific histone modification also found at sites where compensasomes are associated with the male X (Hilfiker et al. 1997; Smith et al. 2000; Akhtar and Becker 2001). Recently, JIL-1, which phosphorylates H3Ser10, was shown to be enriched at the MSL binding sites in males (Wang et al. 2001). Thus, MSL proteins and *roX* RNAs are thought to function in a ribonucleoprotein complex (compensasome) to mediate dosage compensation by altering chromatin structure of the male X chromosome (Stuckenholz et al. 1999; Franke and Baker 2000). In females translational repression of *msl-2* mRNA by the *Sex-lethal* protein (SXL) prevents formation of compensasomes and hence dosage compensation (Bashaw and Baker 1997; Kelley et al. 1997).

The processes and constraints that generate the observed distribution of compensasomes along the male X chromosome are unknown. Although the hundreds of places where compensasomes are found along the X chromosome are referred to as “sites,” they are in fact not points, but rather bands (small segments of chromosome) that roughly span the size range of salivary chromosome bands seen with DNA stains (i.e., a few tens to several hundreds of kilobases in length). Thus, both the locations and the extents of these sites are somehow specified. Furthermore, the compensasome bands do not correspond to the bands where DNA is condensed (Baker et al. 1994; Kelley et al. 1999; Demakova et al. 2003). In addition, non-dosage-compensated X-linked genes (e.g., *LSP1-α*) are scattered throughout the X chromosome and can reside next to dosage-compensated genes (Baker et al. 1994). Since there is no known DNA-binding component in the compensasome, and consensus DNA sequences required for binding have not yet been identified, an understanding of the distribution of compensasomes along the X chromosome needs to encompass not only how complexes are targeted to these several hundred sites, but also how the ends of each band are delimited.

A proposal for how the distribution of compensasome bands along the X chromosome is generated (Kelley et al. 1999) has come from the following findings. MSL-1 and MSL-2 represent core components of the complex: The presence of both is required for either to bind, and none of the other MSL proteins binds to the X chromosome in an *msl-1* or *msl-2* mutant male (Lyman et al. 1997). Furthermore, in males mutant for *mle, msl-3,* or *mof,* binding of MSL-1 and MSL-2 is only maintained at a limited number of sites (35–40) on the X chromosome, which include the *roX1* and *roX2* genes (Lyman et al. 1997; Kelley et al. 1999). Finally, *roX* transgenes inserted into an autosome retain binding of compensasomes, and in addition show compensasome binding in the autosomal region flanking the insertion site, a phenomenon termed spreading (Kelley et al. 1999). Based on these observations, a reasonable model (Kelley et al. 1999) emerged suggesting that the 35–40 sites of MSL-1 and MSL-2 binding on the X seen in *mle, msl-3,* or *mof* mutants represent nucleation sites or entry sites for the complex. From these sites, newly assembled compensasomes would spread in *cis* along the X to form the hundreds of final sites observed in a wild-type male. In this spreading model, *roX* RNAs would also be required for compensasome assembly (Park et al. 2003). However, there is to date no direct evidence that entry sites and spreading play any role in the processes that generate the normal pattern of compensasome binding along the X chromosome. We thus directly tested this model by analyzing various pieces of the X chromosome transposed or translocated to autosomal locations for their ability to bind compensasomes and initiate spreading.

## Results

**Figure 1:**
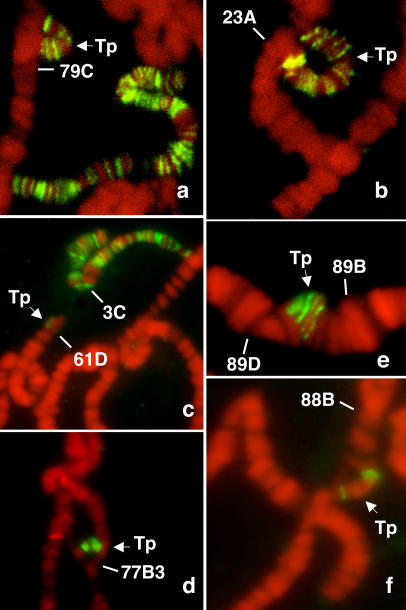
MSL Binding to Pieces of X Chromosome Inserted into Autosomes Salivary glands from males heterozygous for each transposition were fixed (47% acetic acid in phosphate-buffered saline, then lactic acid/water/acetic acid [1:2:3]), squashed on slides, treated with anti-MSL-1 antibodies and a secondary Cy3 anti-rabbit immunoglobulin G antibody, then counterstained with DAPI and viewed using a Zeiss Axiophot microscope. Both duplications and transpositions were able to attract compensasomes, whether or not they contained predicted entry sites. (A) Line II. (B) Line I, which contains the *roX1* gene. (C) Line X shows one to two bands on the smallest transposition we studied; the intensity of the second band was variable even on the X chromosome. (D) Line IV. (E) Line IX. (F) Line XI. Breakpoints (described in Table 1) were verified by cytology when possible and/or with specific probes by in situ hybridization. Gray value images were pseudo-colored and merged.

The spreading model implies that a piece of the X chromosome translocated to an autosome must contain at least one of the 35–40 “entry” sites if that piece of the X is to recruit compensasomes and become dosage compensated. We looked at MSL binding in various chromosome rearrangements that inserted small pieces of X chromosome into autosomal locations. Table 1 summarizes the translocations, transpositions, and duplications examined. The insertions in the first set (lines I to XI) range in size from about 1% to 15% of the length of the X, and the corresponding stretch of X chromosome for each contains 1–19 distinguishable MSL bands. These insertions were examined in heterozygous condition so we could readily identify the junctions between X chromosomal and autosomal material. When large enough, they appear as a loop of unpaired chromosome protruding from the paired autosomes. We found that transpositions containing one (lines VI to VIII) or several (lines I to V) previously described entry sites (Lyman et al. 1997) showed consistent MSL binding along the inserted piece (Table 1; Figure 1A, 1B, and 1D). Surprisingly, transposed pieces of X chromosome lacking any entry site also showed MSL binding when inserted into an autosome (Table 1, lines IX to XI; Figure 1C, 1E, and 1F). For all of these 11 transpositions the binding pattern observed and the intensity of MSL bands reproducibly matched the expected pattern of that piece of the X chromosome in a wild-type male. Even the smallest piece we looked at (line X, approximately 200 kb) showed one to two MSL bands (Figure 1C). Thus, we found that any piece of the X chromosome moved to an autosomal location is able to bind compensasomes, whether or not the transposed piece of X chromosome contains an entry site. This finding suggests that each of the hundreds of MSL bands observed on the X in males carries the information necessary and sufficient to attract compensasomes, and does not require adjacent entry sites.

**Table 1:**
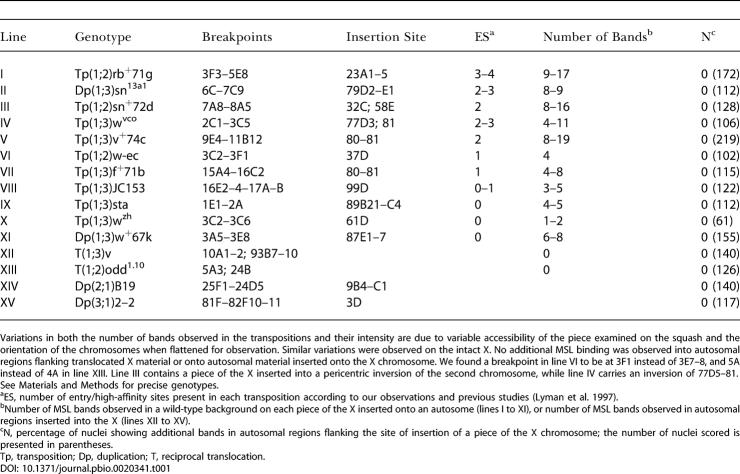
Summary of the Transpositions Studied: Transpositions, Duplications, and Reciprocal Translocations

Variations in both the number of bands observed in the transpositions and their intensity are due to variable accessibility of the piece examined on the squash and the orientation of the chromosomes when flattened for observation. Similar variations were observed on the intact X. No additional MSL binding was observed into autosomal regions flanking translocated X material or onto autosomal material inserted onto the X chromosome. We found a breakpoint in line VI to be at 3F1 instead of 3E7–8, and 5A instead of 4A in line XIII. Line III contains a piece of the X inserted into a pericentric inversion of the second chromosome, while line IV carries an inversion of 77D5–81

See Materials and Methods for precise genotypes

^a^ES, number of entry/high-affinity sites present in each transposition according to our observations and previous studies (Lyman et al. 1997)

^b^Number of MSL bands observed in a wild-type background on each piece of the X inserted onto an autosome (lines I to XI), or number of MSL bands observed in autosomal regions inserted into the X (lines XII to XV)

^c^N, percentage of nuclei showing additional bands in autosomal regions flanking the site of insertion of a piece of the X chromosome; the number of nuclei scored is presented in parentheses

Tp, transposition; Dp, duplication; T, reciprocal translocation

Interestingly, duplications showed binding both along the autosomal insertion and on the X chromosome (lines II and XI), indicating that the supply of compensasomes is not limiting in these circumstances. We also tested homozygous transpositions and duplications for MSL binding in males and found that we could recover MSL binding on each homozygous transposed piece (unpublished data) as well as on the X. Thus, even three copies of the same segment of the X chromosome (two of the duplication plus the original piece on the X) were able to maintain MSL binding. This result extends previous data showing that, by using specific *msl-2* transgenes escaping SXL repression, ectopic expression of MSL-2 in females induced binding to both X chromosomes, in a pattern identical to the single X of a wild-type male (Bashaw and Baker 1997). Therefore, binding occurs regardless of the location and number of copies of the X-linked targeted sequences.

**Figure 2:**
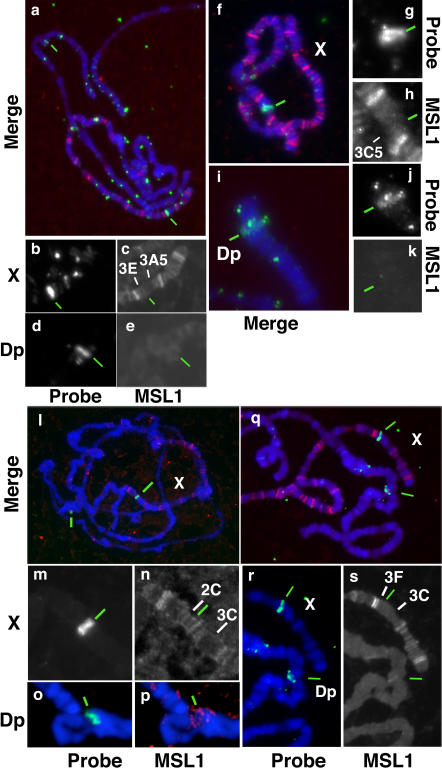
MSL Binding to Autosomal Duplications of X Chromosome Pieces in *mle* or *msl-3* Mutant Larvae Salivary glands from *w*; *pr mle* ^12.17^/*cn bw mle*; *Dp (1;3)/msl2Δ10* or *w; Dp (1;2)/msl2Δ21*; *msl3^p^/msl3^p^* females were squashed and stained as described in Figure 1, followed by in situ hybridization with a biotinylated probe specific for regions carried by each duplication (Lavrov et al. 2004) and incubation with Oregon green-coupled streptavidin. Conditions throughout the procedure were adjusted to maximize MSL staining. Specific biotinylated probes (green bars) appear in green in merges (A, F, I, L, O, Q, and R) and as bright bands in (B, D, G, J, and M). MSL bands are shown in red in merges and in (P) and as bright bands in (C, E, H, K, N, and S). DAPI stain is blue. MSL binding is absent from duplications or the matching region on the X in line XI (3A5–3E8) (A–E) and line X (3C2–3C6) (F–K) in *mle* mutants, confirming that they lack any entry sites. Probe maps region 3D–E in (A–E) and 3C in (F–K). (L–P) Illustrated are the one to three bands detected in *mle* mutant nuclei on the duplicated region from line IV (2C1–3C5) (O and P) and on the same segment on the X (M and N). (O) and (P) are from another nucleus. (Q–S) A single band is detected at the 3F1 breakpoint of the duplication (3C2–3F1, line VI) in *msl-3* mutant nuclei (S), corresponding to the weakest band of the doublet at 3F on the X. Note the weak signal on duplications compared to the same region on the X chromosome. Probe maps region 2D5–3A2 in (L–P) and 3D–E in (Q–S).

The determinations listed in Table 1 of how many entry sites each of the transpositions contains were made by comparing the reported breakpoints of each rearrangement to the described locations of entry sites (Lyman et al. 1997). As cytological determinations can vary, we directly confirmed the presence or absence of entry sites by examining MSL binding in an *msl-3* or *mle* mutant background for a subset of these transpositions (Figure 2). Each line used in these experiments contained the transposed region from the X inserted into an autosome and a wild-type X chromosome. For line XI we found that, in *mle* mutant individuals, MSL binding was undetectable in either the transposed region (3A5–E8) inserted at 87E17 (Figure 2A–2E) or in this region in the wild-type X. As expected, the same is true when only a subset of this region is duplicated: Line X did not show binding in *mle* mutants to region 3C2–3C6 on the X or to the transposition of that region inserted at 61D (Figure 2F–2K). These findings confirm that lines X and XI do not contain entry sites. Similarly, we confirmed that transpositions inferred to contain entry sites in two lines (IV and VI) did in fact contain such sites. Thus, for line IV in an *mle* mutant background we observed MSL binding to one to three sites on both the transposition and the corresponding region of the X (Figure 2N and 2P), while for line VI in an *msl-3* mutant background we observed one site of MSL binding on both the transposition and the corresponding region of the X (Figure 2S). These findings are consistent with those of Lyman et al. (1997), who reported two entry sites in the region encompassed by the transposition in line VI, and one entry site in the region encompassed by the transposition in line IV. Our findings firmly establish that isolated subregions of the X chromosome display normal patterns of compensasome binding irrespective of whether they contain entry sites, and thus suggest that entry sites do not play a distinct role in the establishment of compensasome binding along the X as postulated by the spreading hypothesis. Hereafter we will refer to entry sites as high-affinity sites, their original name (Lyman et al. 1997). During the course of this study, Oh et al. (2004) have reported similar results for binding of compensasomes to transpositions from lines I, VIII, and IX. However, the scale of the analysis and the limited number of rearrangements did not yield the same conclusions.

The two high-affinity sites identified to date correspond to the *roX1* and *roX2* genes (Kageyama et al. 2001; Park et al. 2003), and it was the fact that *roX* transgenes inserted into autosomal locations are able to induce spreading—binding of the MSLs to some autosomal sequences surrounding a *roX* transgene insertion site—that led to the hypothesis that spreading gives rise to the wild-type distributions of compensasome bands along the male X chromosome. We therefore examined whether autosomal transpositions of a piece of the X were able to induce spreading. In cells heterozygous for each of the transpositions listed above we never observed additional MSL binding to the autosomal regions either *cis* or *trans* to the insertion site (Table 1; see Figure 1). We also did not observe additional MSL binding in males homozygous for the transpositions described above. This was true irrespective of the number of high-affinity sites contained in the transpositions. Interestingly, lines I and V, which each contain several high-affinity sites, including the *roX1* or *roX2* gene, respectively, showed no spreading in males wild-type for the MSLs (see Figure 1B). The dichotomy between our results and those obtained with *roX* transgenes suggests that spreading may be a phenomenon restricted to some *roX* transgenes (see below) and not an aspect of dosage compensation.

**Figure 3:**
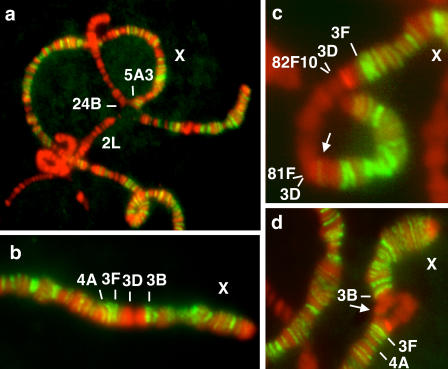
Compensasomes Do Not Spread from the X Chromosome onto Autosomal Regions Inserted on the X (A) Females expressing MSL-2 from an *msl2Δ3–21* transgene and bearing a reciprocal translocation between the X and second chromosome (line XIII) do not show additional bands in the regions of the 2L arm juxtaposed to X chromosome material. (B) MSL binding pattern on the X chromosome of a wild-type male. (C and D) The autosomal region 81F–82F10–11 does not show MSL binding when inserted at 3D in the single X of a male (line XV) (C) or in MSL-2-expressing females heterozygous for the same transposition (D). Note that the MSL binding pattern on the X chromosome is not altered by the insertion. The light band (arrow) maintained on the wild-type unpaired region of the X of a female heterozygous for the transposition is also present next to the same insertion at 3D on the unique X chromosome of a male (compare C and D).

To further assess if spreading in *cis* occurs on the X chromosome, we next asked if the complex could spread from the X onto an autosomal piece attached to the X by a reciprocal translocation. We tested two reciprocal translocations that interchanged large portions of the X and 3R or 2L (see Table 1, lines XII and XIII, respectively). Both translocations separate *roX1* (3F) and *roX2* (10C) genes from one another and thus both pieces of each translocation contain a *roX* locus. Anti-MSL-1 staining revealed the absence of any bands on either of the 3R or 2L pieces of these translocations (Figure 3), while the pattern observed on the two transposed pieces of the X was normal. These results strengthen the idea that spreading may be a phenomenon restricted to *roX* transgenes, since the breakpoints in line XII (10A) and line XIII (5A) are relatively close to the *roX2* (10C) and *roX1* (3F) loci, respectively.

We also tested two small transpositions of autosomal regions into the X (Table 1, lines XIV and XV; Figure 3C): Neither of them showed MSL binding, even weak, to any part of the inserted autosomal sequences. Furthermore, females either heterozygous or homozygous for these transpositions and expressing ectopic MSL-2 did not show any MSL bands in either of these insertions of autosomal material into the X, although they displayed normal MSL binding both to the unpaired X region (in heterozygotes) and along the paired portions of the two X chromosomes (Figure 3D). Thus, insertion of a piece of an autosome into the X does not disrupt MSL binding to either the unpaired X homologue at the insertion site or the regions of the X immediately flanking the site of insertion of autosomal material. Moreover, these results are inconsistent with the model derived from the *roX* transgene studies where MSL binding is observed both in the autosomal regions adjacent to the insertion site and on the wild-type autosomal homologue.

## Discussion

In summary, we have used chromosome rearrangements to test two central aspects of the proposed spreading model of dosage compensation in *Drosophila.* It is worth noting that our experiments were a priori neutral: They could have provided compelling evidence for or against the spreading model. In both cases our results are inconsistent with the clear predictions of that model. First, we show that pieces of the X chromosome inserted into an autosome bind compensasomes in precisely the pattern characteristic of that piece of the X at its endogenous location on the X, and this property is independent of the presence of sites previously described as entry sites. Second, compensasomes do not spread from the X into autosomal pieces inserted into, or translocated onto, the X. Moreover, there is not spreading of compensasomes from autosomal insertions of pieces of the X chromosome into the autosomal regions flanking the insertion, even when such pieces contain a *roX* gene close to the breakpoint. These results suggest that spreading in *cis* is not part of the process of dosage compensation in flies. We thus propose that all of the hundreds of sites along the X chromosome where compensasomes are found in wild-type males are competent to independently recruit compensasomes.

Our findings raise several questions regarding previous data. Are the 35–40 sites that attract partial complexes in *mle* or *msl-3* mutants qualitatively different from the other sites at which MSL bands are found in wild-type, and if so, how? Why do *roX* transgenes induce additional binding to adjacent autosomal sequences?

With respect to the potential heterogeneity of compensasome binding sites, while most of the relevant data are indirect (only the *roX1* and *roX2* genes are identified binding sites), the data are consistent with the simple view that the binding sites are homogeneous in terms of their function, but have varying affinities for compensasomes. Our finding that pieces of X chromosome transposed to autosomal locations display normal patterns of compensasome binding, irrespective of whether or not they contain high-affinity sites, removes the one functional distinction between binding sites that had been proposed. That there are not two classes of binding sites in terms of affinity for compensasomes, but rather a continuum of affinities, is strongly suggested by the recent report of Demakova et al. (2003), who carefully characterized the number and locations of compensasome bands in mutant females expressing various limiting amounts of MSL-2. They found only four bands in the most limiting case, and progressively higher numbers of bands as more MSL-2 protein was expressed. Interestingly, the intermediate 40 sites at which complete complexes are assembled in these conditions exactly matched with the 35–40 high-affinity sites bound by partial complexes in *mle* or *msl-3* mutants. Their data are consistent with a model in which compensasomes continue to bind site specifically to additional sites after all high-affinity sites are occupied, as opposed to spreading from high-affinity sites as previously proposed. Given these findings, a reasonable scenario as to how dosage compensation is achieved would be the following. As MSL expression begins, the high-affinity sites progressively sequester nascent partial or full complexes in the early stages of dosage compensation. When the amount of available complexes or its components increases, sites of higher affinity would accumulate more complexes, while low-affinity sites would remain undetectable, until the former have preferentially assembled sufficient amounts of complexes to make components available for sites with lower affinities. Thus, the compensasomes would progressively bind to different sites along the X according to the different affinities of these sites. Consistent with our model, we found that in *mle* or *msl-3* mutants, duplications maintain binding of partial complexes at the high-affinity sites (Figure 2N, 2P, and 2S), though with a lower affinity than the same site on the X. The latter observation suggests that, in conditions where components of the complex are limiting, binding might also be dependent on the location of these sequences in the cell (see discussion on spreading below).

That compensasome binding sites would have a range of affinities is also consistent with what is known about DNA-binding proteins, which recognize with varying affinities a range of binding sites whose sequences are related to a common consensus. Variations from the consensus can allow temporal and quantitative modulation of individual genes, or subsets of genes. That compensasome binding sites are also likely to vary in sequence, and hence affinities, comes from what is known about sex chromosome evolution in *Drosophila* species (Marin et al. 1996, 2000). During the course of sex chromosome evolution in this genus there are a number of cases in which new X chromosomes have evolved, and in all cases examined to date, this has been accompanied by the new X chromosome gradually acquiring compensasome binding sites as the new Y chromosome, its former homologue, degenerates. The selective advantage of dosage compensation for each gene is determined both by the state of degeneration of the allele on the new Y chromosome and by the degree to which a gene in males requires its function, and thus its expression, to match the output of both wild-type female X chromosomes (Marin et al. 2000). Hence, one would expect individually evolved binding sites to exhibit a range of affinities for compensasomes. Finally, we note that each of the final compensasome bands on the X chromosome displays a reproducible but specific intensity, likely to reflect not only different affinities for compensasomes, but also the length of X chromosome encompassed in each band.

The last issue we wish to address is spreading. The fact that, in chromosome rearrangements that juxtapose pieces of X and autosome, we never observed spreading, even when entry sites or *roX* genes were near the breakpoints, suggests that spreading does not exist naturally on the X chromosome, and is not required to establish the final pattern of binding in *Drosophila* males. Yet spreading from *roX* transgenes is very well documented in a variety of situations. We therefore suggest that spreading is a phenomenon specific to the *roX* transgenes, and a consequence of the key function of *roX* RNAs in dosage compensation. In particular, we propose that the *roX* genes are the sites of assembly of compensasomes using newly synthesized *roX* RNAs, just as the ribosomal RNA genes are the sites where ribosomes are assembled. Thus, *roX* transgenes would generate a high local concentration of compensasomes in their vicinity, competing with other chromatin-binding factors that normally bind to nearby autosomal sequences. In some cases, compensasomes would displace these other factors, resulting in a new compensasome band in the autosomal region flanking the transgene (spreading). Several features of spreading are consistent with this proposal. First, additional bands corresponding to spreading from *roX* transgenes contain *roX* RNA and the H4Lys16 modification, suggesting that they correspond to mature complexes (Kelley et al. 1999). Second, transcription from a *roX* transgene is required to observe spreading of the complex onto neighboring regions (Park et al. 2002, 2003). Third, *roX* transgenes show variable and often no additional bands in a wild-type background, suggesting that spreading is largely dependent on the insertion site and its environment on the autosomes. One possibility would be that these *roX* transgenes lacking spreading are inserted next to sites bound by factors normally counteracting the effect of compensasomes on the autosomes. Such a view is supported by recent data showing that association of compensasomes at some *roX1* transgenes can overcome the effect of methylation-mediated silencers (Kelley and Kuroda 2003). Finally, MSL-1 and MSL-2 co-overexpression leads to mislocalization of partial MSL complexes to the autosomes and the centromere, as well as a dramatic decompaction of the X (Oh et al. 2003), a male-specific phenotype also observed in both *iswi* or *nurf* mutants, two chromatin regulators (Deuring et al. 2000; Badenhorst et al. 2002; Corona et al. 2002). Thus, increasing locally the amount of available complexes can induce new binding of MSL complexes to usually non-dosage-compensated regions.

Molecular studies of dosage compensation in flies, worms, and mammals have revealed some striking similarities between these systems. In all three systems dosage compensation is achieved by a widespread modification of the structure of X chromosome chromatin, and in mammals and flies this involves specific modifications of histones. Dosage compensation in mammals and flies is also similar in that noncoding RNAs are essential components of the dosage compensation machinery. With respect to the other components of the dosage compensation machinery the situation is less clear. While compensasome-related complexes might be present in mammals (orthologs of *msl-1, -2, -3, mle,* and *mof* genes exist in mammalian genomes), some of them have identified functions not related to dosage compensation, and orthologs of *msl-1, -2,* and *-3* were not found in Caenorhabditis elegans (Marin 2003). Up until now it had also been thought that spreading was involved in dosage compensation in all three systems (Park et al. 2002; Oh et al. 2003; Csankoversuszki et al. 2004; Okamoto et al. 2004). However, our findings indicate that in flies each of the bands on the X chromosome at which compensasomes are found in males is able to independently attract those complexes. Thus, at the interband level spreading does not appear to be part of the dosage compensation process in flies. However, it should be noted that our results do not address either how compensasomes are distributed across the tens of kilobases of DNA that likely comprise individual compensasome bands in salivary gland chromosomes, or how that distribution is achieved; it is possible that, at the level of single bands, spreading may be part of the process of dosage compensation.

## Materials and Methods

### 

#### Fly strains and genetic crosses

Flies were raised on standard cornmeal-yeast-agar medium. Fly stocks containing transpositions were obtained from the Bloomington Drosophila Stock Center. Their genotypes are: *Tp(1;2)rb^+^71 g, ct^6^ v^1^/C(1)DX, y^1^ w^1^ f^1^* (line I); *Df(1)ct-J4, In(1)dl-49, f^1^/C(1)DX, y^1^ w^1^ f^1^; Dp(1;3)sn^13a1^/+* (line II); *Tp(1;2)sn^+^72d, f^1^ car^1^/C(1)DX, y^1^ f^1^; Dp(?;2)bw^D^, bw^D^* (line III); *Tp(1;3)w^vco^, v^1^ f^1^: in w^vco^/ClB, B^36d^* (line IV); *Tp(1;3)v^+^74c/FM7a* (line V); *Tp(1;2)w-ec, ec^64d^ cm^1^ ct^6^ sn^3^/C(1)DX, y^1^ w^1^ f^1^* (line VI); *Tp(1;3)f^+^71b/FM6* (line VII); *Tp(1;3)JC153, v^1^/FM7a* (line VIII); *Tp(1;3)sta, sta^1^: ss^sta^/FM3* (line IX); *Tp(1;3)w^zh^, sc^1^ z^1^ w^zh^* (line X); *Df(1)w258–45, y^2^ sn^3^/C(1)DX, y^1^ w^1^ f^1^; Dp(1;3)w^+^67k/+* (line XI); *T(1;3)v, v^A^/FM6* (line XII); *Tp(2;1)odd^1.10^, b^1^ pr^1^ cn^1^ sca^1^/CyO* (line XIII); *Df(2 l)sc19–7/In(2 l)Cy^L^t^R^ In(2R)Cy, Cy^1^ amos^Roi-1^ cn^2^ sp^2^* or *Dp(2;1)B19, y^1^ ed^1^ dp^o2^ cl^1^* (line XIV); *Dp(3;1)2–2, w^1118^; Df(3R)2–2/TM3, Sb^1^* (line XV). Breakpoints and insertion site are referred in Table 1. Some lines contain additional rearrangements referenced in Lindsey and Zimm (1992). Depending on their genotype, each line was crossed to Canton-S males or females for studies of MSL binding in their male progeny. For homozygous transpositions studies, stocks were balanced to give *w; Tp(1;2)/Cyo-GFP* or *w; Tp(1;3)/TM3-GFP* stocks. Non-GFP third instar male larvae were dissected for analysis. For autosome-to-X transpositions, females from lines XIV and XV were mated with *w; msl2Δ3–21/CyoGFP* or *Dp(A;1)/Y; msl2Δ3–21/CyoGFP* males. Non-GFP female larvae were dissected. For *mle* and *msl-3* mutant analysis, stocks were balanced to give *w; Tp(1;2)/CyoGFP; msl3^p^/TM3-GFP* or *w; prmle^12.17^/CyoGFP; Tp(1;3)/TM3-GFP* stocks. Females were crossed to *w; msl3^p^/CyoGFP; msl2Δ3–10/TM3-GFP* or *mle^1^cnbw/CyoGFP; msl2Δ3–21/TM3-GFP* males, respectively. Non-GFP third instar female larvae were dissected for salivary glands polytene chromosomes analysis. Lines expressing MSL-2 from transgenes *msl2Δ3–21* and *msl2Δ3–10* are described in Bashaw and Baker (1995). *Mle* and *msl-3* mutants are described in Fukunaga et al. (1975), Kuroda et al. (1991), and Gorman et al. (1995). All crosses to generate larvae for immunostaining were carried out at 18 °C.

#### Polytene chromosome immunostaining

Glands from male third instar larvae were dissected in PBS/0.7% NaCl, prefixed in 45% acetic acid for 10 s, and then fixed for 2–3 min in lactic acid/water/acetic acid (1:2:3) solution on siliconized coverslips. Glands were squashed and coverslips flipped off after freezing the slides in liquid nitrogen. Slides were then incubated in PBS for 15 min followed by incubation with affinity-purified anti-MSL-1 antibodies (dilution 1:100) as described previously (Gorman et al. 1995). Chromosomes were viewed under epifluorescence optics on a Zeiss Axiophot microscope or a confocal microscope; pictures were taken using Spot software and colored.

#### Immunofluorescent in situ hybridization of polytene chromosomes

Clones RP-98 17.E.2, RP-98 03.D.13, and RP-98 48.O.22 from the Drosophila melanogaster BAC library (BACPAC Resources, Oakland, California, United States) were used to map regions 3D–E, 3C, and 2D5–3A2, respectively. Specific probes were obtained from BAC clone DNA preparations using the Bionick Labelling System (Invitrogen, Carlsbad, California, United States) according to the manufacturer's instructions. Squashes were prepared as described above. Immunostaining with affinity-purified anti-MSL-1 antibodies was followed by incubation with the appropriate biotinylated probe according to the method of Lavrov et al. (2004).

## Supporting Information

### Accession Numbers

The LocusLink (http://www.ncbi.nlm.nih.gov/LocusLink/) accession numbers for the genes and gene products discussed in this paper are *jil-1* (LocusLink 39241), *mle* (LocusLink 35523), *mof* (LocusLink 31518), *msl-1* (LocusLink 35121), *msl-2* (LocusLink 33565), *msl-3* (LocusLink 38779), *roX1* (LocusLink 43963), *roX2* (LocusLink 44673), and SXL (LocusLink 44872).

## Abbreviations

Dp: duplication
*mle*: maleless
*mof*: males on the first
*msl*: male specific lethal
*roX*: RNA on the X
*Sxl*: sex lethal
T: translocation
Tp: transposition
